# Natural killer cells in colorectal cancer liver metastasis: redefining immune surveillance and plasticity

**DOI:** 10.3389/fonc.2026.1975628

**Published:** 2026-10-26

**Authors:** Yi Lu, Chenyue Xia, Jinhui Gu

**Affiliations:** Department of Anorectal Surgery, Suzhou Traditional Chinese Medicine (TCM) Hospital Affiliated to Nanjing University of Chinese Medicine, Suzhou, Jiangsu, China

**Keywords:** cellular plasticity, colorectal cancer, immune surveillance, liver metastasis, natural killer cells, tumor microenvironment

## Abstract

Natural killer (NK) cells are key effectors in innate immunity and serve as early responders against disseminated tumor cells. In colorectal cancer liver metastasis (CRLM), the liver’s unique immune landscape presents both challenges and opportunities for NK cell–mediated tumor control. While NK cells have long been considered uniform cytotoxic sentinels, recent studies have reshaped this view by uncovering a diverse spectrum of NK cell states with distinct migratory, transcriptional, and functional programs. These findings reveal a heterogeneous NK-cell compartment in the metastatic liver shaped by tissue context and microenvironmental cues, with distinct residency-associated and functional programs. Advances in high-dimensional single-cell technologies have accelerated the characterization of NK cell heterogeneity, plasticity, and interactions within the metastatic niche. In this review, we summarize recent insights into the evolving roles of NK cells in CRLM, highlighting how they contribute to immune surveillance, niche remodeling, and potential therapeutic modulation.

## 1 Introduction

Colorectal cancer (CRC) liver metastasis (CRLM) represents a critical bottleneck in the clinical management of CRC and remains a major cause of cancer-related mortality (1). Beyond its anatomical role as a frequent metastatic destination, the liver constitutes a highly specialized immunological organ in which immune tolerance, metabolic regulation, and continuous antigen exposure are tightly integrated. This distinctive immune landscape fundamentally shapes antitumor immunity and creates a unique context in which disseminated tumor cells either succumb to immune surveillance or successfully establish metastatic lesions.

Natural killer (NK) cells are abundant in the liver and are traditionally regarded as frontline effectors of innate antitumor immunity (2, 3). Their capacity to recognize and eliminate transformed cells without prior antigen sensitization has positioned them as central players in early metastatic control. However, accumulating evidence suggests that this canonical view substantially oversimplifies NK cell biology in the metastatic liver. Rather than functioning as a uniform population of cytotoxic sentinels, NK cells exhibit marked heterogeneity in differentiation state, migratory behavior, and effector potential, which are dynamically shaped by the hepatic microenvironment and by evolving tumor-host interactions (4, 5). Recent advances in single-cell and spatially resolved technologies have catalyzed a conceptual shift in our understanding of NK cells during liver metastasis. These studies reveal that NK cells in CRLM undergo context-dependent adaptation, acquiring tissue-resident features, altered receptor landscapes, and specialized functional programs that extend beyond direct cytotoxicity (6). Such heterogeneity and tissue adaptation suggest that NK cells may extend beyond direct cytotoxic surveillance and participate in niche-associated immune regulation, including interactions with tumor, stromal, and immune compartments. Importantly, these findings also expose critical knowledge gaps regarding how NK cell states are established, maintained, or functionally restrained within metastatic lesions (7). Despite rapid progress, current understanding of NK cells in CRLM remains fragmented across disciplines, with insights from liver immunology, tumor biology, and single-cell profiling often considered in isolation. A cohesive framework that integrates NK cell heterogeneity, microenvironmental conditioning, and functional plasticity in the metastatic liver is still lacking. This gap limits not only mechanistic interpretation but also the rational translation of NK cell-based therapeutic strategies to the setting of liver metastasis.

In this review, we synthesize recent advances that redefine NK cell biology in CRLM. We focus on how the hepatic immune landscape shapes NK cell identity and function, how NK cell plasticity influences immune surveillance and niche remodeling through interactions with tumor, stromal, and immune compartments, and how these insights inform emerging therapeutic opportunities. By reframing NK cells as dynamic and context-responsive components of the metastatic microenvironment, this review aims to provide a conceptual foundation for understanding innate immune regulation in CRLM and to highlight avenues for therapeutic modulation of NK cell responses in the liver. Importantly, many current insights into hepatic NK-cell heterogeneity in CRLM are derived from single-cell transcriptomic, spatial profiling, and high-dimensional phenotyping studies. Although these approaches have substantially improved the resolution at which NK-cell states can be characterized, transcriptional or spatial associations do not by themselves establish causal state transitions or functional mechanisms. Throughout this review, we therefore distinguish NK-cell heterogeneity from inferred plasticity and experimentally demonstrated state transitions. We use “heterogeneity” to describe the coexistence of phenotypically or transcriptionally distinct NK populations, “tissue adaptation” to denote context-associated features linked to local residency or conditioning, and “plasticity” to refer to potentially dynamic changes in NK-cell phenotype or function. Importantly, we reserve terms such as “state transition,” “conversion,” or “reversible reprogramming” for settings in which temporal, lineage-tracing, perturbational, or rescue experiments provide direct evidence of change between states.

## 2 NK cell-mediated immune surveillance in homeostasis and early metastatic restraint

NK cells occupy a distinctive position in antitumor immunity: they are pre-armed effectors of the innate immune system that can eliminate transformed cells without prior antigen sensitization (2, 8, 9). Mechanistically, NK cells execute cytotoxicity through perforin/granzyme release and can also engage death-receptor pathways via ligands such as TNFα, FasL, and TRAIL, while simultaneously shaping downstream immunity through cytokine and chemokine production (e.g., IFNγ, IL-10, CCL3/4/5) (10, 11). Beyond soluble mediators, NK-cell activation is determined by the integrated balance of activating versus inhibitory receptor inputs, enabling rapid discrimination of stressed or malignant targets (12, 13). Canonical activating receptors include NKG2D (recognizing stress ligands such as MICA/B and ULBPs) and DNAM-1 (binding CD112 and CD155), while inhibitory receptors such as KIRs and NKG2A enforce self-tolerance by sensing MHC class I or HLA-E, thereby operationalizing “missing-self” recognition when these ligands are reduced on tumor cells (12, 14, 15). In this framework, NK cells are not simply “cytotoxic sentinels” but context-aware decision-makers whose killing, cytokine output, and tissue behavior are continuously tuned by receptor-ligand landscapes.

From a metastasis perspective, NK cells are especially relevant at stages where disseminated tumor cells are exposed and vulnerable. Experimental metastasis models have repeatedly shown that impairing NK function, either by antibody-mediated depletion or by using NK-deficient hosts, leads to increased metastatic outgrowth, consistent with a nonredundant role for NK cells in constraining dissemination and seeding (16). At metastatic sites, innate immune surveillance can also be “licensed” by tissue danger-sensing pathways. In a liver metastasis setting, the Nlrp3 inflammasome-IL-18 axis has been shown to suppress CRC metastatic growth by acting through the innate immune system to promote hepatic NK-cell maturation and to prime Fas-mediated cytotoxicity, providing a concrete example of how local inflammatory circuitry can empower NK tumoricidal activity during metastatic colonization (17). These findings clarify why metastatic tumors often evolve strategies that specifically blunt NK surveillance. One recurring route is to remodel the ligand environment that NK cells “read” through activating receptors. For instance, tumor cells can remodel the activating and inhibitory ligand landscape perceived by NK cells, thereby dampening NK degranulation and cytokine production in co-culture assays-an illustrative paradigm for how altered ligand landscapes shift NK-tumor engagement away from efficient clearance (18). In parallel, tumor metabolic remodeling can further reinforce immune escape; lipid remodeling programs that increase membrane unsaturated phospholipids (e.g., through upregulation of SCD activity) have been associated with reduced susceptibility to NK-mediated lysis and can be partially reversed by pharmacologic or genetic targeting of the lipid pathway, linking metabolic state to NK killing efficiency (19). While these examples are not liver- or colorectal-specific in isolation, they succinctly illustrate a generalizable principle that becomes central in CRLM: metastatic success frequently reflects not the absence of NK cells, but the emergence of tumor- and niche-driven adaptations that reshape the cues governing NK activation, synapse formation, and effector execution.

## 3 Heterogeneity of hepatic NK cells

The liver represents one of the most immunologically complex organs in the body and harbors a uniquely structured NK cell compartment (20). In the context of CRLM, this intrinsic complexity becomes particularly relevant, as NK cells constitute a major innate immune population poised at the interface between immune surveillance and immune tolerance (**Table 1**). Increasing evidence indicates that the effectiveness of NK cell-mediated antimetastatic responses in the liver cannot be understood without first appreciating the marked heterogeneity of hepatic NK cells. Rather than forming a uniform cytotoxic population, liver NK cells comprise multiple subsets with distinct developmental origins, spatial distribution, and functional potential, all of which are shaped by local tissue cues and pathological stress (3, 21). This heterogeneity is not merely descriptive but has direct functional consequences. Different NK subsets vary substantially in their capacity for cytotoxicity, cytokine production, tissue retention, and interaction with other immune or stromal cells. In CRLM, where tumor cells exploit the liver’s tolerogenic tendencies, selective perturbation of specific NK cell states may critically determine whether immune surveillance is maintained or progressively eroded (22). Understanding hepatic NK heterogeneity therefore provides a necessary conceptual framework for interpreting NK cell behavior in metastatic disease.

**Table 1:** Endogenous NK-cell phenotypes and functional programs relevant to colorectal cancer liver metastasis.

NK-cell phenotype /program	Representative markers	Functional characteristics	Microenvironmental drivers	Spatial localization in CRLM	Role in CRLM	Therapeutic relevance	Ref.
Circulating NK cells (cNKs)	CD56dim, CD16, FCGR3A, Eomes	High cytotoxicity, rapid target-cell killing, IFN-γ production	Recruitment through inflammatory chemokines; activation by IL-12, IL-15, IL-18	Liver sinusoids, infiltrating metastatic lesions	Predominantly antitumor surveillance and elimination of disseminated tumor cells	Adoptive NK-cell transfer, cytokine support, trafficking enhancement	(6, 23)
Tissue-resident NK cells (trNKs)	CXCR6, CD69, CD49a, CD56 bright	Tissue retention, cytokine production, local immune regulation	Hepatic residency signals, chronic antigen exposure, local cytokine conditioning	Liver parenchyma and metastatic niche interface	Context-dependent; may support immune surveillance or tissue-adapted regulation	Modulation of tissue-residency programs and local activation pathways	(21, 22)
CD49a^+^/ILC1-like tissue-associated phenotypes	CD49a, CXCR6, CD69 (variable Eomes/T-bet profiles)	Reduced migratory capacity, tissue adaptation, specialized effector programs	TGF-β signaling, chronic tissue conditioning, stromal interactions	Peritumoral and intratumoral regions	Context-dependent; antitumor-associated programs have been identified in CRLM, whereas tumor-permissive CD49a^+^ phenotypes are supported primarily by other hepatic tumor settings	State-specific intervention; functional restoration for reversible restraint versus selective targeting of stable tumor-permissive populations	(5, 24, 25)
Activated effector NK programs	NKG2Dhigh, DNAM-1high, IFN-γhigh, Granzyme B high	Enhanced cytotoxicity and immune surveillance	IL-12, IL-15, IL-18, inflammatory signaling	Regions with active immune infiltration	Strong antitumor activity	Cytokine agonists, NK engagers, engineered NK therapies	(6, 17)
Functionally restrained NK programs	Variable NKG2A expression; TIGIT, KIRs, altered activating/inhibitory receptor balance	Reduced cytotoxicity, impaired synapse formation, limited effector responses	Chronic suppressive signaling, hypoxia, adenosine, metabolic stress, TGF-β	Established metastatic lesions	Associated with impaired immune surveillance and metastatic persistence	Checkpoint blockade, cytokine support, metabolic restoration, or niche-targeted therapy when functional restraint is reversible	(6, 22, 26)
Metabolically constrained NK programs	Variable phenotype; impaired RhoA signaling and cytotoxic programs	Defective immune synapse formation, reduced tumor-cell killing	Carnitine-mediated suppression, nutrient competition, hypoxia	Metabolically stressed metastatic regions	Tumor-permissive due to impaired effector function	Targeting BBOX1-carnitine axis, metabolic restoration approaches	(27, 28)

These categories are not mutually exclusive. Circulating and tissue-resident designations primarily describe cellular compartmentalization, whereas CD49a^+^/ILC1-like phenotypes reflect tissue-associated features and activated, restrained, or metabolically constrained programs describe functional properties that may overlap across NK-cell populations.

CRLM, colorectal cancer liver metastasis; cNK, conventional/circulating NK cell; trNK, tissue-resident NK cell; IFN-γ, interferon-γ.

### 3.1 Tissue-resident versus circulating NK cell compartments in the liver

A central organizing principle of hepatic NK cell heterogeneity is tissue residency. The liver hosts both circulating NK cells that traffic through the sinusoidal vasculature and tissue-resident NK cells that are locally maintained within the hepatic niche (29). Tissue-resident NK cells are characterized by the expression of retention- and homing-associated molecules, including CXCR6 and CD69, reflecting their long-term adaptation to the liver microenvironment (30, 31). These cells are shaped by continuous exposure to local cytokines, metabolic signals, and cell-cell interactions, resulting in transcriptional and functional profiles distinct from those of circulating NK cells.

In the setting of CRLM, disruption of this tissue-resident compartment emerges as a recurring feature. The CXCR6^+^ CD69^+^ NK population is markedly diminished within metastatic lesions, suggesting that tumor growth interferes with the maintenance of resident NK niches or actively excludes these cells from the tumor-bearing liver (22). Importantly, tissue-resident NK cells are not simply redundant with circulating NK cells; rather, they appear to fulfill non-overlapping roles in hepatic immune defense (23). Experimental studies indicate that effective antimetastatic immunity in the liver can involve the coordinated activity of conventional NK cells (cNKs) and tissue-resident group 1 innate lymphoid cells (trILC1s), highlighting complementary roles within the group 1 innate lymphoid compartment (23). This layered organization of hepatic NK immunity has important implications for CRLM. Recruitment of circulating NK cells alone may be insufficient to compensate for the loss or dysfunction of tissue-resident subsets. Consequently, quantitative assessments of NK cell infiltration in metastatic livers may fail to capture qualitative defects in immune surveillance if residency status and functional specialization are not taken into account.

### 3.2 Defining tissue-resident NK cells and ILC1-like populations: classification challenges

A major challenge in interpreting hepatic NK-cell heterogeneity is the lack of a universally accepted boundary between tissue-resident NK cells and ILC1-like populations (32). Although conventional circulating NK cells are generally distinguished by their migratory behavior and cytotoxic effector phenotype, tissue-adapted NK-lineage cells in the liver often share overlapping markers and transcriptional features (3). This overlap is particularly evident for molecules associated with tissue retention, including CD49a, CXCR6, and CD69, which are frequently used to define resident or ILC1-like compartments but are not exclusive to a single lineage state (33). In mice, liver ILC1s are commonly described as CD49a^+^ DX5^-^ cells with strong tissue residency, whereas conventional NK cells are often characterized as DX5^+^ Eomes^+^ populations with greater circulatory potential (34). In humans, however, these distinctions are less straightforward. Markers such as CD49a, CXCR6, CD69, CD56, and CD16 are variably used across studies, and their expression may change in response to inflammation, tumor exposure, or local cytokine cues (35). Consequently, populations described as tissue-resident NK cells, liver-resident NK cells, CD49a^+^ NK cells, or ILC1-like NK cells may partially overlap depending on the species, tissue context, experimental platform, and gating or clustering strategy used. This nomenclature issue is especially relevant in CRLM, where metastatic lesions can reshape NK-cell phenotypes and blur conventional lineage boundaries. For instance, CD49a expression may indicate tissue adaptation and spatial retention, but it does not by itself define whether a population represents a stable ILC1 lineage, a resident NK-cell state, or a tumor-conditioned NK program (5, 34). This distinction is important because lineage identity, tissue adaptation, and functional state are biologically related but not interchangeable concepts. Lineage heterogeneity refers to developmentally distinct populations, whereas tissue adaptation describes phenotypic and transcriptional features associated with prolonged residence in a particular microenvironment. Functional states, in contrast, may reflect more transient changes in activation, metabolism, or effector competence. Cross-sectional single-cell or spatial datasets can distinguish these phenotypes but generally cannot determine whether one population has transitioned into another. Demonstration of true state conversion requires longitudinal tracking, lineage tracing, or controlled perturbation showing movement between defined states. Similarly, CXCR6 and CD69 support tissue residency but should be interpreted together with developmental markers, transcription factors, spatial localization, and functional readouts (21). Therefore, when discussing hepatic NK subsets in CRLM, it may be more accurate to use state-based terminology, such as “tissue-resident NK cells,” “ILC1-like NK cells,” or “CD49a^+^ tissue-adapted NK states,” while acknowledging that these categories may not be fully interchangeable across studies. Recognizing this classification ambiguity is important for interpreting apparently divergent findings in the literature. Studies that report protective or tumor-promoting roles of CD49a^+^ or tissue-adapted NK populations may not necessarily be examining identical cell types. Differences in marker panels, tumor models, species, and analytical resolution may contribute to variable conclusions. A more standardized framework integrating surface phenotype, transcriptional identity, developmental features, spatial localization, and functional validation will be essential for defining hepatic NK-lineage states in CRLM more precisely. This distinction is not merely semantic: therapeutic interpretation depends on whether a phenotype reflects a stable lineage-defined population or an environmentally conditioned functional state that may be amenable to reversal.

### 3.3 Phenotypic remodeling of NK cells within CRLM lesions

Beyond residency, hepatic NK cells undergo extensive phenotypic remodeling in response to the metastatic microenvironment. Within CRLM lesions, NK cells are reduced in overall abundance compared with non-tumor liver tissue, but this reduction is accompanied by a selective reshaping of NK subpopulations. Notably, there is a relative enrichment of CD56^dim^ CD16^+^ NK cells, suggesting a shift toward subsets traditionally associated with peripheral circulation rather than tissue adaptation (6). At the receptor level, CRLM-associated NK cells exhibit a profound imbalance between activating and inhibitory signaling pathways. The activating receptor NKG2D, a key mediator of tumor cell recognition, is markedly downregulated, while the inhibitory receptor NKG2A is upregulated (5, 22). In parallel, increased expression of NKp44 further illustrates the complexity of receptor rewiring in the metastatic setting (22). Rather than indicating a single process such as activation or exhaustion, this altered receptor landscape identifies a distinct phenotypic state associated with modified signaling thresholds and effector potential. Whether these features represent stable adaptation, transient remodeling, or a reversible transition cannot be determined from cross-sectional phenotyping alone. Transcriptional regulation may add another layer to this phenotypic remodeling. Studies outside the CRLM setting have demonstrated that lineage-defining transcription factors such as T-bet critically regulate NK-cell differentiation and antimetastatic function, providing a mechanistic framework for interpreting transcriptional changes observed in tumor-associated NK cells (36). However, direct evidence linking altered T-bet activity to NK-cell dysfunction in human CRLM remains limited. Thus, the receptor and transcriptional changes observed in CRLM should currently be interpreted primarily as evidence of phenotypic remodeling rather than proof of a specific causal differentiation program. Together, these observations reinforce a critical concept: the presence of NK cells within metastatic lesions does not equate to preserved antitumor function. Instead, NK cells in CRLM occupy distinct phenotypic states that may actively limit their cytotoxic potential while allowing their numerical persistence.

### 3.4 Single-cell resolution of functional NK states and spatial heterogeneity

High-dimensional single-cell and spatial approaches have provided unprecedented resolution of NK-cell heterogeneity in human CRLM. These studies identify transcriptionally distinct NK-cell populations rather than a simple linear activation gradient. Importantly, the coexistence of these transcriptional states demonstrates heterogeneity but does not by itself establish that NK cells dynamically transition between them. Among them, ILC1-like CD49a^+^ populations have been associated with strong cytotoxic and antitumor transcriptional programs within metastatic lesions (5). Importantly, such transcriptional signatures indicate functional potential but do not, in isolation, establish superior tumor-cell killing or causal protection against metastasis. Their biological relevance is strengthened when considered together with spatial information, receptor expression, and functional evidence from complementary experimental systems. Chemokine signaling, notably via CXCR3, contributes to NK-cell localization within CXCL9- and CXCL10-rich tumor-associated regions (5). In experimental CRC liver-metastasis models, metastasis-infiltrating NK cells were shown to co-localize with macrophages in these chemokine-rich niches, supporting a functional relationship between local myeloid composition and NK-cell positioning (5). The direct correspondence of these findings to spatial NK-cell organization in human CRLM requires further validation. Whether such interactions reinforce cytotoxic programs or impose regulatory constraints likely depends on the balance of signals within the metastatic microenvironment. These findings highlight that hepatic NK heterogeneity in CRLM is multidimensional, encompassing phenotypic, functional, and spatial diversity. Antitumor NK activity is concentrated within specific cellular states and anatomical niches rather than uniformly distributed across the NK compartment. This insight has important implications for both mechanistic interpretation and therapeutic intervention, emphasizing the need to consider NK cell state and localization when evaluating immune surveillance in CRLM. These observations define candidate antitumor-associated NK programs in CRLM but should not be interpreted to mean that residency-associated phenotypes are uniformly protective across tumor contexts, an issue discussed below.

### 3.5 Dual functionality of hepatic NK states in CRLM

Although NK cells are classically regarded as antitumor effectors, their biological significance in the metastatic liver cannot be reduced to a simple protective-versus-dysfunctional dichotomy (7). In human CRLM, recent single-cell and spatial studies have identified NK-cell populations with distinct tissue-associated, migratory, and cytotoxic transcriptional programs, including CD49a^+^/ILC1-like populations enriched for antitumor effector signatures (5, 6). These observations support substantial functional heterogeneity within the hepatic NK-cell compartment, although transcriptional state alone does not establish either lineage identity or direct antimetastatic activity. Importantly, superficially similar tissue-associated phenotypes can have different biological meanings in different tumor contexts. In hepatocellular carcinoma, for example, accumulation of tumor-infiltrating CD49a^+^ NK cells has been associated with impaired antitumor immunity and unfavorable clinical outcome (24). Such observations should not be interpreted as evidence that an equivalent tumor-promoting CD49a^+^ population exists in CRLM. Rather, they demonstrate that residency-associated markers such as CD49a do not encode a fixed functional identity and that the consequences of tissue adaptation depend on lineage background, local cytokine exposure, tumor type, and spatial context. More direct evidence for tumor-permissive NK-cell activity in CRLM comes from experimental liver-metastasis models. A recent study demonstrated that phenotypically resting NK cells can facilitate colon cancer liver metastasis by increasing tumor-derived stem cell factor (SCF), revealing an indirect route through which NK cells may support metastatic growth without simply losing all effector capacity (37). This finding is conceptually distinct from the CD49a^+^ populations identified by single-cell profiling and should not be taken to indicate that all tissue-adapted or low-cytotoxicity NK states are protumoral. Instead, it illustrates that NK cells can participate in metastatic regulation through mechanisms extending beyond direct target-cell killing. These apparently divergent observations also raise an important distinction between stable lineage programs and environmentally conditioned functional states. Some hepatic group 1 innate lymphoid populations may represent developmentally established tissue-resident lineages with relatively stable transcriptional identities, whereas other tumor-associated NK phenotypes may emerge through cytokine-, metabolic-, or stromal-dependent conditioning (25, 38). In the latter case, functional restraint may remain at least partially reversible if the responsible environmental signal is removed or therapeutically disrupted. In contrast, strategies aimed at a stable lineage-defined population would need to consider selective recruitment, expansion, depletion, or replacement rather than assuming that functional reprogramming alone will restore cytotoxicity. This distinction has direct therapeutic implications. A broadly activating strategy could be beneficial when antitumor NK cells are present but functionally restrained, yet potentially counterproductive if a tumor-permissive NK population is simultaneously expanded or preserved. Conversely, indiscriminate depletion of tissue-associated NK cells could remove subsets that contribute to local tumor control. Therapeutic intervention should therefore be guided by the identity, functional program, and reversibility of the targeted NK population rather than by a single surface marker or by total NK-cell abundance. This principle is consistent with emerging evidence from other tumor settings showing that NK cells can function not only as direct cytotoxic effectors but also as context-dependent immune regulators, underscoring the potential risk of therapeutically manipulating the NK compartment without resolving functionally distinct subsets. In this framework, checkpoint blockade, cytokine support, metabolic restoration, or disruption of suppressive niche signals may be most appropriate for reversible functional restraint, whereas stable lineage-associated programs may require more selective cellular strategies. Collectively, current evidence supports a context-dependent model in which hepatic NK-cell populations can contribute to either antitumor surveillance or tumor-permissive regulation, but the balance between these functions depends on tumor context, lineage identity, spatial localization, and microenvironmental conditioning. Defining these variables more precisely will be essential before apparently opposing NK-cell phenotypes can be translated into reliable therapeutic targets in CRLM.

## 4 NK cell state remodeling and microenvironmental conditioning in CRLM

Hepatic NK-cell heterogeneity in CRLM is closely associated with microenvironmental conditioning, but the extent to which this heterogeneity reflects true cellular plasticity remains incompletely resolved. Single-cell, spatial, and phenotypic studies demonstrate the coexistence of NK populations with distinct receptor profiles, transcriptional programs, metabolic features, and tissue-associated phenotypes. These findings are consistent with substantial context-dependent remodeling but do not, by themselves, establish lineage conversion or reversible state transitions. We therefore use “plasticity” in this section to describe experimentally supported or strongly inferred changes in NK-cell phenotype and function, while distinguishing these from stable lineage differences or tissue-resident programs. In CRLM, the metastatic microenvironment may favor particular NK-cell phenotypes through altered cytokine signaling, metabolic stress, spatial restriction, and prolonged cellular interactions, thereby reshaping the functional composition of the NK compartment (**Figure 1**).

**Figure 1:**
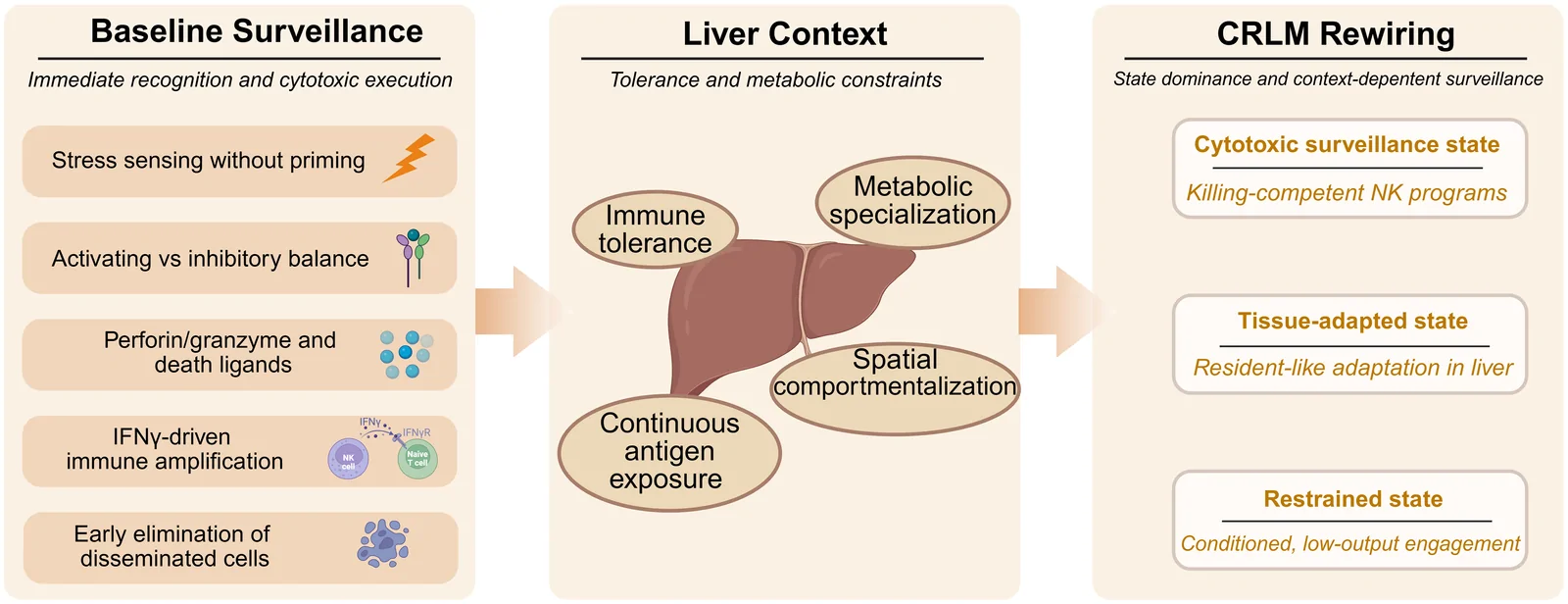
Rewriting NK immune surveillance in CRLM: from uniform killers to state-based control. NK cells provide rapid innate immune surveillance by integrating activating and inhibitory signals to execute cytotoxic programs and coordinate downstream immunity. In the liver, however, immune tolerance, metabolic specialization, continuous antigen exposure, and spatial compartmentalization reshape NK differentiation and function. In CRLM, NK responses are therefore best understood through a state-based framework in which distinct NK states coexist and immune control depends on which functional programs dominate and where they operate within metastatic lesions.

### 4.1 Microenvironmental signals shaping NK cell states in the metastatic liver

The metastatic liver microenvironment exposes NK cells to a sustained array of signals that are associated with altered NK cell identity and functional state. Tumor-derived factors, altered cytokine landscapes, and prolonged interactions with resident hepatic cells collectively remodel NK cell functional programs (39). Importantly, these signals do not induce uniform suppression of NK cells. Instead, they bias NK cells toward specific phenotypic states, selectively reinforcing tissue-adapted or regulatory features while attenuating robust cytotoxic responses (7). Evidence from experimental CRLM models demonstrates that immune surveillance in the liver is highly sensitive to shifts in innate immune signaling circuits. For example, disruption of inflammasome-dependent signaling profoundly alters hepatic NK cell maturation and tumoricidal capacity (17). Activation of the NLRP3 inflammasome promotes IL-18-dependent licensing of NK cells, enhancing FasL expression and tumor cell killing, whereas loss of this pathway results in functionally immature NK cells and accelerated metastatic growth (17). These findings highlight that NK cell competence in the metastatic liver is actively maintained by microenvironmental signals rather than being intrinsically hardwired. Conversely, studies in broader liver-metastasis models indicate that pathological reprogramming of the hepatic immune milieu can impose strong suppressive pressure on innate cytotoxic lymphocytes (40). For example, hepatocyte-intrinsic activation of cell cycle-related kinase signaling can promote an immunosuppressive hepatic environment characterized by myeloid-derived suppressor-cell accumulation and reduced innate cytotoxic surveillance (40). Although this mechanism has not been established as a general feature of human CRLM, it provides a mechanistic precedent for how liver-derived signals can indirectly compromise NK-cell-mediated metastatic control. Together with CRLM-specific evidence for inflammasome-dependent NK licensing, these findings support a model in which local immune competence reflects the balance between activating and suppressive signals within the metastatic liver. This balance can alter the relative representation and function of cytotoxic versus restrained NK phenotypes, linking broader niche remodeling to impaired immune surveillance.

### 4.2 Metabolic and spatial constraints as drivers of NK cell reprogramming

Beyond immune signaling pathways, metabolic and spatial constraints within the metastatic liver can shape NK-cell phenotype and functional competence (41). As a central metabolic organ, the liver imposes stringent energetic and biosynthetic demands on resident immune cells. Metastatic outgrowth further perturbs local nutrient availability and metabolic flux, creating conditions under which NK cell effector functions-closely coupled to cellular metabolic state-become selectively constrained (42). More broadly, experimental studies of NK-cell immunometabolism demonstrate that cytotoxic competence is tightly coupled to cellular metabolic fitness and inflammatory licensing (43). These studies provide a mechanistic basis for interpreting metabolically restrained NK-cell phenotypes observed in tumor-bearing tissues, but they should not be taken as direct evidence that a single metabolic program explains NK dysfunction in CRLM. In the metastatic liver, metabolic stress is therefore best viewed as one component of a broader set of conditions that may constrain NK-cell effector activity, alongside cytokine availability, spatial access, and interactions with surrounding cells (44). Spatial organization within the liver further reinforces NK cell state specialization. Sinusoidal architecture, endothelial barriers, and compartmentalized chemokine gradients dictate where NK cells can localize, persist, and interact with tumor or accessory cells (45). Spatially restricted immune niches may favor tissue-associated NK phenotypes while limiting the access or persistence of highly cytotoxic populations, adding a spatial dimension to NK-cell functional heterogeneity in CRLM.

### 4.3 Plasticity versus dysfunction: redefining NK cell immune surveillance in CRLM

A central challenge in interpreting NK-cell biology in CRLM is distinguishing phenotypic heterogeneity from tissue adaptation, plasticity, and persistent dysfunction. These categories describe different biological phenomena and should not be used interchangeably. Heterogeneity refers to the coexistence of phenotypically, transcriptionally, or functionally distinct NK populations at a given time point. Tissue adaptation describes features associated with prolonged residence or conditioning within the hepatic microenvironment, including altered receptor expression, metabolic programs, and tissue-retention markers. Neither phenomenon alone demonstrates that individual NK cells have transitioned between states.

Plasticity, in contrast, implies that NK-cell phenotype or function can change in response to environmental perturbation. Strong evidence for plasticity therefore requires more than cross-sectional differences between cell clusters. Longitudinal tracking, lineage-tracing approaches, controlled exposure to defined cytokines or metabolic conditions, withdrawal of the conditioning stimulus, or genetic and pharmacological perturbation followed by restoration of phenotype or function can provide evidence that a state is dynamically acquired and potentially reversible. Studies across tumor models have shown that cytokine and growth-factor signaling can reprogram NK-cell effector and regulatory phenotypes, supporting the broader concept of TME-driven NK-cell plasticity (7, 25); however, equivalent reversibility has not yet been comprehensively demonstrated in human CRLM. Dysfunction should likewise be interpreted cautiously. Reduced cytotoxicity, altered receptor expression, metabolic impairment, or limited synapse formation indicate functional restraint, but they do not necessarily define terminal exhaustion. A functionally restrained state may remain reversible if removal of suppressive cues or restoration of activating, metabolic, or cytokine signals recovers effector competence. By contrast, designation of a terminally dysfunctional state would require evidence that impaired function persists despite relief of the initiating microenvironmental constraint. Current CRLM data rarely meet this threshold. We therefore interpret many NK phenotypes observed in CRLM as context-associated functional states rather than fixed endpoints. Some may reflect stable tissue-resident or lineage-defined programs, whereas others may represent environmentally conditioned states with the potential for reversibility. Distinguishing these possibilities will require longitudinal sampling, lineage-resolved analyses, ex vivo functional recovery experiments, and perturbational studies in clinically relevant CRLM models. This distinction is therapeutically important because reversible functional restraint may be amenable to checkpoint blockade, cytokine support, metabolic restoration, or niche-targeted intervention, whereas stable lineage programs may require different strategies focused on recruitment, replacement, or selective expansion.

## 5 NK cell interactions within the metastatic niche

In CRLM, NK cell behavior is not determined solely by cell-intrinsic programs but is profoundly shaped by sustained interactions with tumor cells, myeloid populations, and stromal elements within the metastatic niche. These interactions form a dynamic regulatory network that governs NK cell positioning, functional state, and long-term fate. Importantly, the metastatic niche does not simply suppress NK cells in a uniform manner; instead, it selectively rewires NK cell communication pathways, reshaping immune surveillance through context-dependent crosstalk.

### 5.1 NK-tumor cell interactions: from immune recognition to adaptive evasion

Direct interactions between NK cells and metastatic CRC cells constitute an important checkpoint of immune surveillance in the liver. During metastatic seeding, disseminated tumor cells encounter hepatic NK cells in a microenvironment that can support stress-induced ligand recognition and cytotoxic engagement (40). Under these conditions, NK cells retain the capacity to eliminate vulnerable tumor cells before stable metastatic outgrowth is established. In established metastatic lesions, however, available experimental and cross-sectional evidence indicates that NK-tumor interactions may become less conducive to productive cytotoxic responses and more compatible with tumor-cell persistence (40). This difference should not be interpreted as direct evidence of a longitudinal transition of individual NK cells during human CRLM progression. Rather, it is consistent with context-dependent remodeling of the NK-tumor interface, including altered activating and inhibitory signaling landscapes within the metastatic niche. Tumor cells can modify the cues perceived by NK cells, thereby recalibrating signaling thresholds and reshaping the consequences of cell-cell contact without necessarily excluding NK cells from the lesion. Importantly, experimental models of liver metastasis demonstrate that this loss of effective NK-mediated tumor control is not inevitable but depends on the integrity of local innate immune licensing circuits. When inflammasome-dependent pathways remain intact, NK cells undergo IL-18-dependent maturation and maintain FasL-mediated cytotoxicity, effectively constraining metastatic growth in the liver (17). Disruption of this axis results in functionally immature NK cells and loss of early tumoricidal control, highlighting that NK-tumor interactions are dynamically regulated by the surrounding immune context rather than fixed at the time of tumor arrival.

### 5.2 NK-myeloid crosstalk as a dominant regulatory axis

Within the metastatic liver, NK cells are embedded in dense myeloid networks that exert sustained regulatory pressure on their functional state. Kupffer cells, infiltrating macrophages, and suppressive myeloid subsets shape the immune microenvironment in which NK cells operate, influencing activation thresholds, survival signals, and effector persistence (46–48). In this setting, NK cell behavior reflects not only intrinsic programming but also continuous instruction by surrounding myeloid populations. Evidence from experimental liver-metastasis models illustrates how hepatocyte-driven myeloid reprogramming can indirectly alter innate antimetastatic immunity. In one such model, activation of CCRK-dependent NF-κB signaling induced CXCL1 expression and promoted PMN-MDSC recruitment, establishing an immunosuppressive hepatic niche permissive to metastatic growth (49). Depletion of these suppressive myeloid populations reduced metastatic burden, supporting a causal role for the myeloid circuit in that experimental setting (49). Although this pathway has not yet been established as a defining mechanism of human CRLM, it provides a relevant mechanistic precedent for how changes in hepatic myeloid composition may influence NK-cell competence. Such findings support a model in which NK-myeloid crosstalk sets immune “set points” rather than enforcing absolute suppression. By continuously adjusting the balance between activating and restraining signals, myeloid populations bias NK cells toward tissue-adapted or regulatory states that are compatible with metastatic persistence (**Figure 2**).

**Figure 2:**
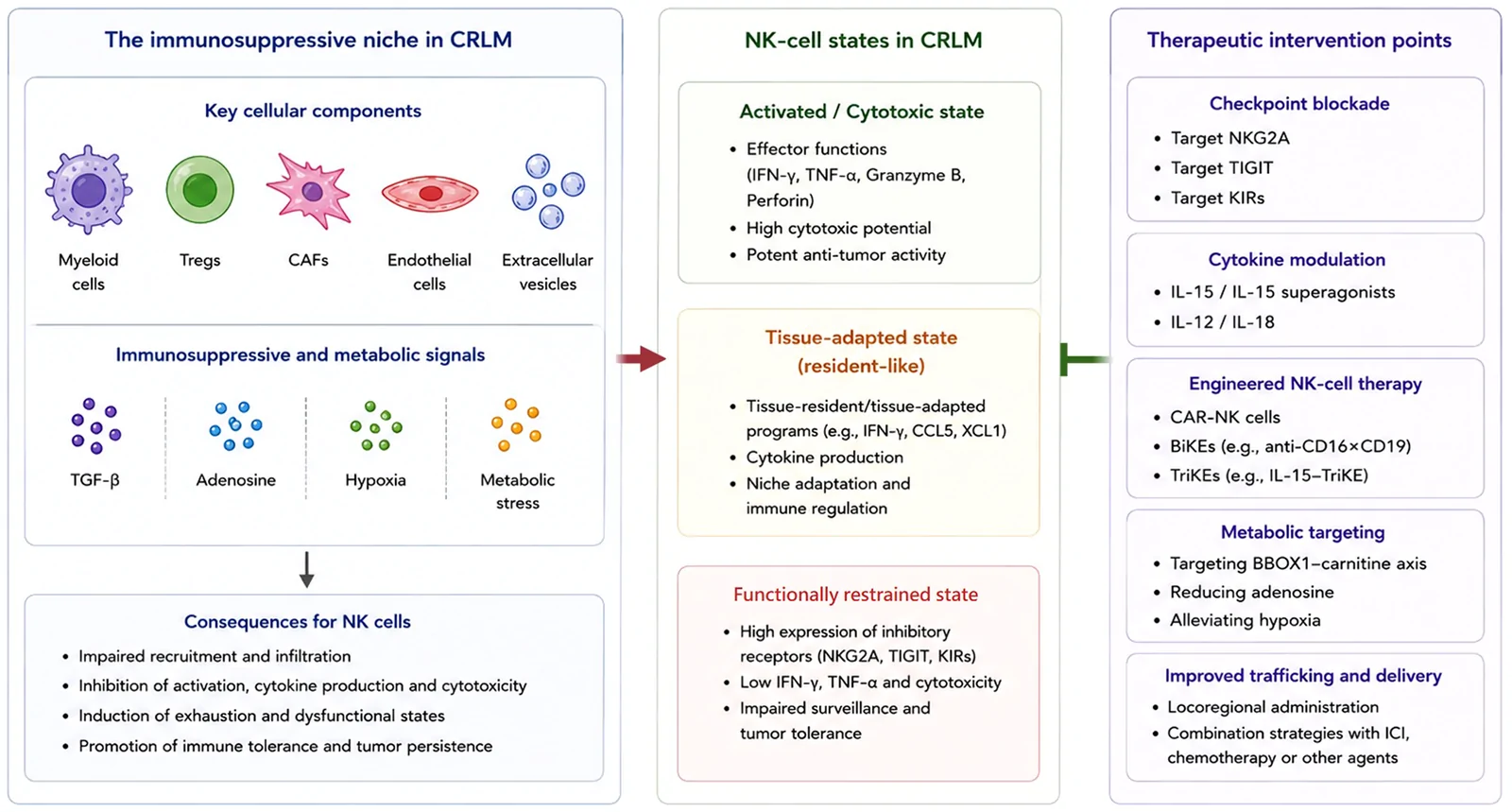
Regulation of NK-cell states and therapeutic intervention points in CRLM. The metastatic liver microenvironment continuously conditions NK-cell behavior through coordinated cellular and metabolic signals. Interactions with myeloid populations, regulatory T cells, cancer-associated fibroblasts, endothelial cells, and extracellular vesicles, together with suppressive factors such as TGF-β, adenosine, hypoxia, and metabolic stress, shape NK-cell differentiation and function within metastatic lesions. As a result, NK cells adopt distinct functional states ranging from activated cytotoxic programs to tissue-adapted and restrained states with reduced surveillance capacity. This framework highlights that immune control in CRLM is determined not simply by NK-cell abundance but by the dominant NK-cell states that emerge within the metastatic niche. Therapeutic intervention can therefore be directed toward both restoring NK effector function and disrupting the microenvironmental signals that maintain suppressive NK-cell states.

### 5.3 Interactions with stromal and endothelial components

Beyond immune-immune interactions, stromal and endothelial compartments impose spatial and kinetic constraints that critically shape NK cell function in CRLM. The sinusoidal architecture of the liver, together with endothelial activation during metastasis, determines when and where immune cells encounter disseminated tumor cells (50). These physical features influence not only immune cell trafficking but also the timing of immune surveillance relative to tumor cell extravasation. Endothelial cells emerge as active participants in this process (51). Liver sinusoidal endothelial cells respond to immune-derived cues by altering vascular permeability, thereby modulating tumor cell extravasation into the parenchyma. In experimental liver-metastasis models, CD4^+^ T-cell-derived IL-22 was shown to promote angiogenesis within established metastatic lesions, partly through endothelial IL-22 signaling, thereby supporting metastatic growth (52). These findings highlight that endothelial cells can actively shape the metastatic niche beyond their structural role in the hepatic vasculature. Such stromal and endothelial constraints spatially compartmentalize NK cell activity. Even when cytotoxic NK states are present, restricted access or delayed engagement can limit their impact, reinforcing localized immune regulation rather than uniform tumor control across the metastatic lesion.

Taken together, tumor, myeloid, stromal, and endothelial signals converge to shape NK-cell function within the metastatic niche. Some of these constraints may be reversible, although direct evidence in human CRLM remains limited. This interdependence provides a rationale for targeting selected tumor–immune or stromal–immune interaction axes rather than focusing exclusively on NK-cell activation.

### 5.4 Multilayered suppressive circuits restraining NK-cell surveillance in CRLM

While myeloid populations represent a major regulatory axis in CRLM, NK-cell dysfunction within metastatic liver lesions is unlikely to be explained by myeloid suppression alone. The hepatic metastatic niche contains multiple overlapping suppressive circuits that collectively limit NK-cell infiltration, activation, and cytotoxic execution. These circuits arise from regulatory immune cells, stromal remodeling, extracellular vesicle-mediated communication, and metabolic stress, creating a microenvironment in which NK cells are progressively shifted away from effective tumor surveillance. Several of these suppressive mechanisms are well established in cancer immunology but remain incompletely validated specifically in CRLM. Regulatory T cells can suppress NK-cell activity through inhibitory cytokines and competition for immune-supportive signals, whereas cancer-associated fibroblasts and other stromal populations can generate physical barriers and immunoregulatory mediators that restrict NK-cell access or function (53, 54). Evidence for these interactions derives largely from broader tumor settings and therefore provides a mechanistic framework for CRLM rather than direct proof of identical circuits in human liver metastases. Tumor-derived extracellular vesicles are particularly relevant to CRC liver metastasis because they can condition the hepatic microenvironment before overt colonization (55). However, direct evidence linking individual CRC-derived vesicular signals to specific NK-cell state transitions remains limited (56). Similarly, hypoxia, adenosine, and TGF-β are established suppressors of NK-cell effector function across multiple tumor systems (26, 28). Their presence in the metastatic liver makes them plausible contributors to NK-cell restraint in CRLM, but the relative importance of each pathway in human CRLM has not yet been resolved. These observations therefore support a multifactorial model in which CRLM-specific mechanisms coexist with suppressive pathways inferred from broader tumor immunology. TGF-β represents a central suppressive mediator linking stromal activation, immune tolerance, and NK-cell reprogramming. By altering NK-cell receptor expression, maturation, and effector potential, TGF-β-associated signaling can shift NK cells toward less cytotoxic and more tissue-adapted phenotypes (57). Together, these findings indicate that impaired NK-cell surveillance in CRLM is unlikely to arise from a single suppressive pathway, but instead reflects the combined influence of immune, stromal, vesicular, and metabolic constraints within the metastatic niche.

## 6 Redefining immune surveillance and therapeutic implications in CRLM

The emerging view of NK cell heterogeneity, plasticity, and niche-level interactions necessitates a reassessment of immune surveillance in CRLM (**Table 2**). Classical models conceptualize immune surveillance as a balance between effector cell abundance and cytotoxic capacity, implicitly assuming that restoring immune cell numbers or activating canonical killing pathways is sufficient to re-establish tumor control. However, accumulating evidence from CRLM challenges this paradigm, suggesting that immune surveillance in the metastatic liver cannot be explained by NK cell presence alone, but should also be interpreted in relation to specific NK cell states shaped by microenvironmental conditioning.

**Table 2:** Evidence hierarchy for NK-cell biology and therapeutic translation in colorectal cancer liver metastasis.

Evidence context	Study/model	NK-cell feature or pathway	Principal finding	Evidence level and limitation	Translational implication	Ref.
Human CRLM	Resected human CRLM; immune profiling	NK-cell infiltration	Higher intratumoral NK-cell infiltration is associated with improved survival	Clinical association; does not establish causality or treatment prediction	Candidate prognostic biomarker	(58)
Human CRLM + experimental CRLM	Human CRC single-cell data integrated with experimental CRC liver-metastasis models	CXCR3-associated NK programs and CD49a^+^/ILC1-like phenotypes	Human metastatic samples support distinct NK-cell programs, while experimental models demonstrate CXCR3-dependent NK-cell localization and macrophage-associated positioning	Mixed human descriptive and preclinical mechanistic evidence; causal spatial and functional relationships were established primarily in experimental models	Supports state- and spatially informed biomarker development, but mechanistic findings require direct validation in human CRLM	(5)
Experimental CRLM	CRC liver-metastasis model	NLRP3–IL-18–FasL axis	Inflammasome signaling promotes NK maturation and antimetastatic cytotoxicity	Functional perturbation supports causality in a preclinical CRLM setting	Cytokine-licensing pathways as therapeutic targets	(17)
Experimental CRLM	CRC liver-metastasis model	Resting NK cell–SCF axis	A resting NK-cell population can indirectly support metastatic progression through tumor-derived SCF	Preclinical mechanistic evidence; human relevance requires validation	Context-dependent targeting of tumor-permissive NK programs	(37)
Experimental metastasis model	Spontaneous/metastatic tumor model	BBOX1–carnitine–RhoA axis	Tumor-derived carnitine disrupts NK-cell synapse formation and immune killing	Mechanistically validated but not established specifically in human CRLM	Metabolic restoration and combination with adoptive NK therapy	(27)
Other liver malignancy	Human HCC	CD49a^+^ NK phenotype	CD49a^+^ NK accumulation is associated with impaired antitumor immunity and poor outcome	Disease-context-specific evidence; should not be directly extrapolated to CRLM	Illustrates context dependence of tissue-adapted NK phenotypes	(24)
Other solid tumors/platform studies	Preclinical and early clinical studies	NKG2A, TIGIT, KIR, BiKE/TriKE	NK-directed interventions can enhance antitumor activity in selected settings	Limited direct CRLM evidence	Therapeutic precedent for future CRLM trials	(59, 60)
Metastatic CRC, non-hepatic dominant disease	Phase 1 locoregional CAR-NK study	NKG2D CAR-NK	Feasibility, tolerability, and preliminary antitumor activity observed	Small uncontrolled study in peritoneal metastasis; does not establish CRLM efficacy	Supports further testing of engineered NK approaches in site-specific CRC trials	(61)

CRLM, colorectal cancer liver metastasis; CRC, colorectal cancer; NK, natural killer; ILC1, group 1 innate lymphoid cell; NLRP3, NLR family pyrin domain containing 3; IL-18, interleukin-18; FasL, Fas ligand; SCF, stem cell factor; BBOX1, γ-butyrobetaine hydroxylase 1; RhoA, Ras homolog family member A; HCC, hepatocellular carcinoma; NKG2A, natural killer group 2 member A; TIGIT, T cell immunoreceptor with Ig and ITIM domains; KIR, killer-cell immunoglobulin-like receptor; BiKE, bispecific killer cell engager; TriKE, trispecific killer cell engager; CAR-NK, chimeric antigen receptor natural killer cell; NKG2D, natural killer group 2 member D.

### 6.1 A state-based framework for immune surveillance in the metastatic liver

Before considering how NK-cell states influence immune surveillance, it is important to recognize that CRLM develops within one of the most immunologically restrictive metastatic environments. Compared with several other metastatic sites, colorectal liver metastases are frequently characterized by limited lymphocyte infiltration, weak spontaneous antitumor immune activation, and extensive immune tolerance (45). The unique hepatic microenvironment, which evolved to maintain tolerance toward dietary and microbial antigens delivered through the portal circulation, creates substantial barriers to effective antitumor immunity (62). Consequently, immune-cell recruitment, activation, and persistence are often constrained within metastatic lesions. These features have important implications for immunotherapy. Despite major advances in immune-based treatments across multiple tumor types, responses in CRLM remain comparatively limited, particularly in microsatellite-stable disease (63). The combination of restricted immune infiltration, suppressive stromal and myeloid networks, and metabolic conditioning contributes to a niche in which endogenous immune surveillance is frequently insufficient to achieve durable tumor control (53, 64). Understanding how NK-cell states are shaped within this context is therefore essential not only for interpreting immune failure in CRLM but also for identifying strategies capable of overcoming liver-specific barriers to immunotherapy.

Immune surveillance in CRLM cannot be explained by NK-cell abundance alone. Clinical and experimental observations indicate that NK cells may remain detectable within metastatic lesions despite ineffective tumor control, suggesting that their functional state and spatial positioning are equally important. We therefore view NK-mediated surveillance as a balance among functionally distinct populations whose activity is shaped by the hepatic microenvironment. In this framework, metastatic progression may reflect a relative shift away from effective cytotoxic programs toward tissue-adapted or functionally restrained phenotypes.

### 6.2 Therapeutic rewiring of NK states: from boosting activation to targeting conditioning circuits

The evidentiary basis for these therapeutic approaches is heterogeneous. Whereas some mechanistic targets have been validated in experimental CRC liver-metastasis models, most NK-directed interventions have been developed in broader solid-tumor settings and remain insufficiently tested specifically in CRLM. Current NK-oriented approaches in metastatic CRC generally fall into two categories: those that aim to strengthen NK cytotoxic function, and those that seek to relieve the suppressive conditioning imposed by the metastatic niche (65, 66) (**Table 3**). Across these studies, a consistent observation is that NK-mediated tumor control is not dictated solely by NK cell infusion or activation, but by whether NK cells can maintain productive tumor engagement under metastatic niche constraints. In this context, inhibitory checkpoint pathways represent a central therapeutic entry point. NKG2A and TIGIT regulate NK-cell activation thresholds by limiting responses to tumor cells that retain inhibitory ligands or exploit altered receptor-ligand balance (59, 60). Therapeutic blockade of these axes is therefore designed to restore NK-cell responsiveness rather than merely increase NK-cell abundance. For CRLM, where tumor-infiltrating NK cells often display altered activating and inhibitory receptor profiles, these checkpoint-directed approaches provide a rational strategy to restore NK-cell responsiveness and relieve functionally restrained phenotypes. Accordingly, the goal of checkpoint modulation should be to restore function within demonstrably restrained antitumor NK populations rather than to indiscriminately amplify all NK-cell states present in the metastatic niche. Cytokine-based NK modulation provides another layer of therapeutic intervention. IL-15 and IL-15 superagonists can enhance NK-cell expansion, survival, and cytotoxicity, whereas IL-12 and IL-18 promote effector activation and functional maturation (67). This is particularly relevant to liver metastasis, given the role of inflammasome-dependent IL-18 signaling in supporting NK maturation and FasL-associated antimetastatic activity (17). However, cytokine stimulation alone may not be sufficient if suppressive niche signals persist, suggesting that cytokine-based strategies may be most effective when integrated with approaches that relieve inhibitory or metabolic constraints. Metabolic rewiring has also emerged as a promising direction for restoring NK-cell competence. Tumor-derived metabolic programs can impair NK-cell cytotoxicity, receptor signaling, and immunological synapse formation. The identification of BBOX1-driven carnitine production as a mechanism that suppresses NK-cell RhoA activity and disrupts synapse formation illustrates how metabolic checkpoints can directly disable NK-mediated killing (27). These findings suggest that targeting tumor metabolic programs or restoring NK metabolic fitness may complement adoptive NK-cell transfer and other NK-activating therapies. Finally, combination strategies may be required to translate NK-cell activation into durable control of metastatic disease. Chemotherapy can increase tumor immunogenicity and stress-ligand expression in some settings, while immune checkpoint inhibitors may relieve adaptive immune suppression and reshape the broader tumor immune environment (68). Combining these approaches with NK-directed therapies may improve both direct cytotoxic engagement and downstream immune amplification (69, 70). In CRLM, such combinations should be designed with attention to liver-specific barriers, including immune tolerance, stromal restriction, and limited immune-cell infiltration.

**Table 3:** NK-directed therapeutic strategies relevant to CRLM.

Therapeutic strategy	Principal target/mechanism	Current evidence context	Potential relevance to CRLM	Major limitation	Ref.
Inhibitory-receptor blockade	NKG2A, TIGIT, KIR	Clinical/preclinical evidence mainly outside CRLM	Restore responsiveness of restrained antitumor NK populations	CRLM-specific clinical efficacy not established	(59, 60)
Cytokine support	IL-15, IL-12, IL-18	Preclinical and broader clinical development	Enhance NK survival, maturation and effector function	Systemic toxicity and persistent niche suppression	(17)
Metabolic targeting	BBOX1–carnitine–RhoA and related metabolic constraints	Mechanistic preclinical evidence	Restore immunological synapse formation and killing competence	Human CRLM validation limited	(27)
BiKE/TriKE	Tumor antigen–NK-cell engagement	Predominantly preclinical/early clinical platform evidence	Improve tumor recognition and contact-dependent killing	Limited CRLM-specific evidence	(73)
CAR-NK	Engineered tumor recognition	Early clinical evidence in metastatic CRC and other cancers	Potential adoptive NK platform for CRLM	Hepatic trafficking, persistence, and CRLM efficacy remain unresolved	(61)
Trafficking/homing strategies	Chemokine-receptor engineering	Preclinical platform evidence	Improve localization of NK cells to liver metastases	Limited clinical validation	(5, 74)
Locoregional delivery	Regional administration of NK-cell therapies	Early clinical precedent outside CRLM	Increase local effector-cell exposure	Benefit and hepatic distribution in CRLM not established	(61)
Combination approaches	NK therapy plus checkpoint, cytokine, chemotherapy or niche modulation	Mostly preclinical/early clinical evidence	Address simultaneous activation and microenvironmental restraint	Optimal combinations and patient selection unknown	(70)

CRLM, colorectal cancer liver metastasis; NK, natural killer; NKG2A, natural killer group 2 member A; TIGIT, T cell immunoreceptor with Ig and ITIM domains; KIR, killer-cell immunoglobulin-like receptor; IL-15, interleukin-15; IL-12, interleukin-12; IL-18, interleukin-18; BBOX1, γ-butyrobetaine hydroxylase 1; RhoA, Ras homolog family member A; BiKE, bispecific killer cell engager; TriKE, trispecific killer cell engager; CAR-NK, chimeric antigen receptor natural killer cell.

An important consequence of NK-cell heterogeneity is that therapeutic activation should not be assumed to have uniformly beneficial effects across the entire NK compartment. If reduced effector function reflects reversible checkpoint-, cytokine-, or metabolism-dependent restraint, strategies that restore activation may recover antitumor competence. In contrast, if a tumor-associated population represents a stable tissue-adapted or lineage-defined program with regulatory or tumor-permissive activity, nonspecific expansion or activation may be ineffective or potentially counterproductive. Therapeutic design should therefore distinguish restoration of function from alteration of cellular composition. The former may be achieved through checkpoint blockade, cytokine support, or metabolic and niche-directed interventions, whereas the latter may require selective recruitment, depletion, replacement, or engineered cellular approaches.

Beyond classical inhibitory receptors, several immunoregulatory pathways may further shape NK-cell dysfunction in liver metastasis. IL-1R8/SIGIRR functions as a negative regulator of IL-18-dependent NK-cell maturation and effector activity, thereby linking innate cytokine licensing to NK-cell competence in the liver (71). Chemokine receptor programs, including CXCR4-associated trafficking pathways, may also influence NK-cell positioning by retaining immune cells within specific chemokine-defined compartments and limiting their access to tumor-relevant niches (6). In addition, TGF-β1 represents a central suppressive signal in the hepatic metastatic microenvironment, capable of altering NK-cell receptor expression, maturation state, and cytotoxic potential (25). Together with NKG2A, TIGIT, and KIR pathways, these regulatory axes indicate that NK-cell dysfunction in CRLM is controlled by a layered network of inhibitory receptors, cytokine checkpoints, chemokine positioning signals, and stromal-derived suppressive cues.

At the mechanistic level, recent work has revealed that metastatic tumor cells can evade NK surveillance by targeting the physical execution of cytotoxicity rather than broadly suppressing immune infiltration (72). A metabolism-focused CRISPR screen performed in a spontaneous metastasis model identified BBOX1 expression in a subpopulation of tumor cells as a determinant of immune evasion (27). In this setting, BBOX1-driven carnitine production inhibited RhoA activity in NK cells, thereby preventing immunological synapse formation and shielding metastatic cells from NK-mediated killing. Functionally, genetic loss of BBOX1 in tumor cells promoted NK-dependent destruction *in vitro* and, importantly, improved the efficacy of NK adoptive transfer therapy *in vivo*. In parallel, experimental studies in liver metastasis models further indicate that NK competence is sustained by defined innate licensing circuits rather than being constitutively fixed. In a CRLM setting, activation of the NLRP3 inflammasome was shown to restrain metastatic growth through an IL-18-dependent program that promotes NK cell maturation and primes FasL-mediated cytotoxicity, highlighting that NK-mediated control can be reinforced by preserving inflammatory licensing cues. Conversely, disruption of this axis resulted in functionally immature NK cells and accelerated metastatic progression, illustrating that loss of specific conditioning inputs can be sufficient to collapse antimetastatic NK function even in the presence of NK infiltration (17). These observations support a therapeutic logic shift: effective intervention in metastatic disease should prioritize restoring state stability and execution competence (e.g., synapse formation and killing modality) while simultaneously counteracting the conditioning circuits that bias NK cells toward restrained programs. In CRLM, this implies that NK-based therapies may benefit from integration with strategies that target metabolic immune evasion, preserve licensing cytokine pathways, and prevent rapid reconditioning within the liver metastatic niche. Thus, these approaches should currently be viewed as mechanistically supported therapeutic opportunities rather than established NK-directed treatments for CRLM, with the strength of evidence varying considerably across individual targets.

### 6.3 Translational opportunities: engineered NK therapies and locoregional delivery paradigms

Clinical development of engineered NK-cell therapies has accelerated across hematologic malignancies and solid tumors, whereas direct clinical evidence in CRLM remains scarce. In metastatic CRC, emerging studies provide early evidence of feasibility and immune modulation, although most have not specifically enrolled patients with liver-dominant disease. CAR-NK platforms and bispecific or trispecific NK-cell engagers should therefore currently be regarded as emerging translational strategies rather than established therapies for CRLM. In addition to CAR-NK platforms, bispecific and trispecific NK-cell engagers (BiKEs and TriKEs) have expanded the therapeutic toolkit for redirecting NK-cell cytotoxicity (73). These molecules are designed to bridge NK cells with tumor-associated antigens while simultaneously engaging activating receptors or cytokine-supportive modules, thereby enhancing immune synapse formation, tumor recognition, and NK-cell persistence (73). Although their application in CRLM remains at an early stage, the conceptual relevance is clear: in a metastatic niche where productive NK-tumor engagement is frequently impaired, engager-based strategies may provide a flexible approach to restore contact-dependent killing without requiring permanent cellular engineering. A second translational challenge is the limited trafficking and persistence of NK cells in solid tumors (75). For liver metastases, anatomical barriers, sinusoidal architecture, stromal remodeling, and chemokine mismatch can restrict NK-cell access to tumor foci (5). Strategies that improve hepatic homing, chemokine-guided migration, and local persistence are therefore likely to be essential for effective NK-based therapy in CRLM (74). These may include engineering chemokine receptors, combining NK-cell therapy with niche-remodeling agents, or using locoregional delivery routes to increase effector-cell concentration at metastatic sites. Importantly, several NK-cell checkpoint pathways discussed above have already entered clinical development across solid tumors and hematologic malignancies. Therapeutic strategies targeting NKG2A, KIRs, TIGIT, and related NK-regulatory axes are being evaluated either as monotherapies or in combination with immune checkpoint blockade, cytokine support, adoptive NK-cell transfer, or engineered NK platforms (59). Although CRLM-specific trials remain limited, these studies provide important translational precedent for targeting inhibitory NK-cell circuits and support the incorporation of NK-focused immune monitoring into future liver metastasis trials. A notable example is a phase 1 dose-escalation study evaluating intraperitoneal NKG2D CAR-NK-cell administration in heavily pretreated patients with colorectal cancer and peritoneal metastases (61). Among nine treated patients, three achieved stable disease, providing preliminary evidence of clinical activity alongside the reported feasibility and tolerability of locoregional CAR-NK delivery. Multi-omics and functional analyses further suggested that CAR-NK-cell infusion can influence endogenous adaptive immunity, including expansion of activated cytotoxic CD8^+^ T-cell populations. Importantly, these findings should not be interpreted as direct evidence of efficacy in CRLM. The study involved peritoneal rather than liver metastases, and intraperitoneal administration does not establish effective hepatic trafficking or antitumor activity within the liver metastatic niche. Nevertheless, the study provides a useful translational precedent for engineered NK-cell therapy in metastatic CRC and illustrates the potential of locoregional cellular therapy to combine direct cytotoxicity with broader immune modulation. Whether similar approaches can overcome the distinct trafficking, stromal, metabolic, and immunoregulatory barriers of CRLM will require dedicated liver-metastasis studies. Collectively, engineered NK-cell platforms and locoregional delivery strategies represent emerging directions in metastatic CRC. Their application to CRLM will require specific solutions for hepatic trafficking, spatial access, persistence, and resistance to liver-specific suppressive cues.

### 6.4 Clinical relevance, biomarkers, and translational challenges

Clinical evidence supports the relevance of NK-cell immunity in CRLM, but its translation into prognostic or predictive frameworks remains incomplete. In resected colorectal liver metastases, increased intratumoral NK-cell infiltration has been associated with improved overall survival, particularly when accompanied by higher T-cell infiltration (58) (**Table 2**). Recent studies further indicate that CRLM is biologically heterogeneous, with distinct immune and stromal ecosystems characterized by differences in lymphocyte infiltration, immune activation, stromal organization, and therapeutic responsiveness (53). These differences are likely to influence not only NK-cell abundance but also their localization and functional state, with important implications for patient stratification and the development of NK-directed therapies. Integrating spatial immune profiling, single-cell analysis, and functional biomarkers may therefore help identify biologically distinct CRLM subgroups and provide a more informative framework than NK-cell enumeration alone.

Importantly, prognostic associations should not be conflated with predictive biomarkers. In patients undergoing hepatectomy after neoadjuvant chemotherapy, higher intratumoral NK-cell and T-cell infiltration was independently associated with improved overall survival, supporting the prognostic relevance of coordinated cytotoxic lymphocyte infiltration (58). However, predictive biomarkers of response to NK-directed therapy have not yet been validated in CRLM. Future biomarker strategies will likely need to integrate NK-cell abundance with functional and spatial features, including NKp46^+^ infiltration, tissue-resident or CD49a^+^ NK populations, activating and inhibitory receptor profiles, metabolic restraint signatures, and spatial proximity to tumor, myeloid, or stromal compartments. Such biomarkers should also resolve functional directionality, because phenotypically similar tissue-associated populations may support antitumor surveillance in one context while accompanying immune-regulatory or tumor-permissive programs in another. Candidate predictive features include activating and inhibitory ligand expression, chemokine programs that support NK-cell recruitment, and metabolic states that impair immune-synapse formation or effector competence (6).

Clinical translation must also account for heterogeneity beyond the NK-cell compartment. Most CRLM is microsatellite stable, whereas the smaller MSI-H/dMMR subgroup has a distinct immune context and greater sensitivity to immune checkpoint blockade (63). Treatment history should also be considered when interpreting NK-cell phenotypes in CRLM. Prior systemic therapy may influence NK-cell abundance and functional competence (58). By contrast, the effects of liver-directed interventions on the local NK-cell compartment remain insufficiently defined. Accordingly, studies evaluating NK-directed approaches should report molecular subtype and treatment history and, where feasible, incorporate paired or longitudinal sampling before and after systemic or liver-directed therapy.

A further challenge is that many mechanistic insights into NK-cell regulation in liver metastasis derive from preclinical models. Although these studies have identified candidate pathways involving inflammasome–IL-18 licensing, trafficking, metabolic restraint, and immune-synapse formation, their clinical relevance requires validation in human CRLM. Translation will therefore depend on reproducible biomarker panels that integrate NK-cell phenotype, function, and spatial organization; standardized terminology for tissue-resident, ILC1-like, and functionally restrained NK populations; and validated spatial and functional assays that can be applied reproducibly across centers. Prospective CRLM cohorts with longitudinal sampling will be essential to determine whether these biomarkers are associated with recurrence, treatment response, or survival. Ultimately, NK-state biomarkers should be incorporated prospectively into clinical trial design, enabling biomarker-defined patient stratification and direct evaluation of predictive value.

## 7 Conclusion

NK cells are central components of immune surveillance in CRLM, but their contribution cannot be understood from abundance or cytotoxic potential alone. The metastatic liver contains heterogeneous NK-cell populations shaped by tissue residency, spatial organization, metabolic conditions, and interactions with tumor, myeloid, and stromal compartments. These influences generate distinct functional programs that may support antitumor surveillance or, in some contexts, accompany tumor-permissive regulation. Importantly, tissue-associated phenotypes or reduced cytotoxicity should not be assumed to represent either fixed dysfunction or protumoral activity; their biological significance depends on lineage identity, microenvironmental context, and the degree to which functional restraint is reversible.

These insights have important translational implications. Restoring NK-mediated control in CRLM will likely require more than increasing NK-cell numbers or applying nonspecific activation. Therapeutic strategies should instead consider the functional identity, spatial localization, and microenvironmental constraints of the targeted NK population. Progress toward this goal will depend on improved state definition, validation of clinically relevant biomarkers, and prospective testing of NK-directed approaches in CRLM-specific cohorts.
